# To Correspondents

**Published:** 1880-12

**Authors:** 


To Correspondents.—All communications intended for publication in this Journal must be accompanied by the real name of the writer, otherwise they cannot be inserted, however meritorious they may be. It is not intended that the name of the author shall appear, when it is not desired it should, but to be kept for any reference to it that might be required. Whilst we do not assume responsibility for the opinions or utterances of anonymus contributors, their real names will be known only to us. Great latitude and freedom will be allowed such correspondents ; on *all* subjects looking to the advancement and welfare of the profession.

